# Mechanical performance of graphene_x_/poly(ether ketone ketone) composite sheets by hot pressing

**DOI:** 10.1038/s41598-022-08221-0

**Published:** 2022-03-08

**Authors:** Q. B. Wang, D. L. Jia, X. H. Pei, X. L. Wu, F. Xu, Z. H. Ye, H. X. Wang

**Affiliations:** 1 Department of Oil & Gas Production Equipment, Research Institute of Petroleum Exploration and Development, Xueyuan Road 20#, Beijing, 100083 China; 2 School of Mechanical Engineering, Jiangsu University, Xuefu Road 301#, Zhenjiang, 212001 China

**Keywords:** Engineering, Materials science, Physics

## Abstract

Polymer composites are gradually replacing traditional metal materials in the fields of aviation, aerospace, automotive and medicine due to their corrosion resistance, light weight and high strength. Moulding technology and organization morphology of polymer composite are key elements affecting the quality of products and their application, so a vacuum hot pressing process for graphene_x_/poly(ether ketone ketone) (PEKK) (x = 0%, 2%, 3%, 4%, 5%, 6%) composite powders is explored with particularly designed moulding parameters to achieve high conductive properties and good mechanical properties in graphene/PEKK composite sheet with thickness of 1.25 mm and diameter of 80 mm. The vacuum environment ensures that the graphene is not oxidized by air during hot pressing molding, which is essential for achieving conductive property in the graphene/PEKK composite; The hot pressing temperature of each graphene/PEKK composite powder is higher than glass transition temperature but lower than melting temperature, which ensures the graphene/PEKK composite powders is fully compacted and then graphene is fully lapped in the composite sheet. In addition, the graphene/PEKK composite sheet shows conductive property when the graphene content increases to 3wt%, and then the conductivity of the composites increases and then decreases with a peak value at 5wt% with increasing graphene content. By comparing the mechanical properties and microstructure morphology of the graphene/PEKK composite sheets, it was obtained that graphene content has an obvious effect on the mechanical properties of the composites, e.g., the mechanical properties will be increased as the graphene content increasing when graphene content is more than 3%. The graphene distribution law of the composite material with different graphene contents is analysed using a scanning electron microscope (SEM).

## Introduction

Recently, with the rapid development of materials science, computer science, mathematics and physics, polymer composites are gradually replacing some traditional metal materials to be applied in aviation, aerospace, automotive and medical applications because of their unique mechanical and chemical properties^1^. The different elements, structures, combinations and forming methods of polymer composite materials determine the mechanical, physical and chemical properties of polymer composite products. According to this feature, researchers have modified the polymer composite materials to obtain different properties and realize different applications. The polyaryletherketone^2,3^ group of polymer products is a widely used product in aerospace and oilfield, usually forming polymer composites with metals and organic and inorganic substances. Polyaryletherketone matrix composites have been developed to meet the demands of various applications^4^.

The Poly(ether ketone ketone) (PEKK) and Poly(ether ketone ketone) PEEK are the well-known members of the PAEK family^5–8^. PEEK is more popular than PEKK both in field of scientific research and the real engineering as polymer matrix of composite. While, in addition to similar mechanical properties and crystalline behaviour to PEEK, PEKK received considerable attention because of its low price, low melting point and stable process, which results in better molding performance for PEKK composite and more applications^9^ as compared to PEEK composite. However, PEKK is insulator, which limited its application in many fields, such as electronic engineering field. Given its resistance to corrosion, conductivity, lightweight, versatility and simple process, graphene is used as a filler material, which is dispersed into the PEKK matrix to produce a new composite material and replace traditional metal materials, and then its application range has been further expanded^10^.

Although the preparation of polymer composite materials is a prerequisite for a good product, the moulding process is an indispensable condition for a good product. Moulding determines the quality and additional performance of the product. For the thermoplastic composite material, traditional forming technology includs injection moulding, hot pressing moulding and extrusion injection moulding. Injection moulding is an important polymer processing method^11^. Injection moulding can achieve complex shapes for thermoplastic composite material, and it has good material flowability, high production efficiency and good dimensional accuracy. While, it is limited to high fluidity of liquid, which is hard for PEKK matrix, even in a high temperature above its melting temperature. Extrusion injection moulding^12^ is a highly efficient, continuous, low-cost forming technology for polymeric composites, but the cost of equipment and moulds is high. With the development of additive manufacturing^13,14^, 3D printing technology^15,16^ has been developed to a large extent, and it is gradually applied to the medical field, printing human organs, etc. While the formed materials are poor in density, in addition to demanding requirements to the size of row material powder particles, which greatly limits the application of 3D printing technology for polymeric composites.

In constrast to above forming methods, hot pressing process is a simple and widely used processing method in the plastic processing industry, especially for thermoplastic polymer matrix composites. The factors that affect the material state during hot pressing process are mainly the composition of the polymer composite, the pressure on the moulding, the ambient temperature and atmosphere environment, etc^17–19^. Donadei revealed that residual stresses formed during hot pressing process for carbon fiber-reinforced PEKK powder determined most of the defects during heating, and an annealing treatment was proposed to reduced the formation of these defects^20^. Alsadon processed PEKK polymer using a standard ceramic pressing furnace and aimed to optimize the hot pressing parameters in order to achieve optical and mechanical properties of pressed samples^21^. Fujihara prepared CF/PEEK composite by hot pressing process with a molding temperature around melting temperature of PEEK, and the experimental results indicated higher hot pressing temperature led to decomposition of the PEKK matrix^22^. It is obvious that the design molding parameters according to raw material powder and the final performance is crucial for hot pressing molding. However, there is relative less research on GR/PEKK composite process, especially on its hot pressing molding^23^.

In this paper, we aimed to explore a vacuum hot pressing process for molding of graphene_x_/PEKK (x = 0%, 2%, 3%, 4%, 5%, 6%) composite powders in order to achieve conductive graphenex/PEKK composite sheet with good mechanical properties with a focus on design of molding parameters. In addition, the effect of graphene content on the conductivity, mechanical performances and corresponding microscopic morphology of the composite sheets were investigated.

## Materials and methods

### Materials

Given the poor dispersibility of graphene in the PEKK matrix based on the traditional solution dispersion and melt blending in the preparation of polymer composite materials, graphene/PEKK conductive composite powder was prepared by situ polymerization to make graphene nano particles uniformly dispersed in the PEKK matrix, which has been presented in our previous work^23^. Table 1 shows the size and conductivity of PEKK and graphene/PEKK composite powder particles.

**Table 1:** The size and conductivity of PEKK and graphene/PEKK composite powder particles.

Composites	Length (μm)	Width (μm)	Conductivity of graphene and composite sheet (S/cm)
PEKK	150–306	75–220	–
GR_MG1_/PEKK	100–220	55–140	(50–100)/1 × 10^–7^
GR_MG2_/PEKK	729	414	(50–100)/–
GR_MG3_/PEKK	120–180	50–70	(2000–3000)/1 × 10^–4^
GR_MG4_/PEKK	150–200	80–130	(2000–3000)/1 × 10^–7^
GR_MG5_/PEKK	100–300	70–120	(1000)/–

**Figure 1:**
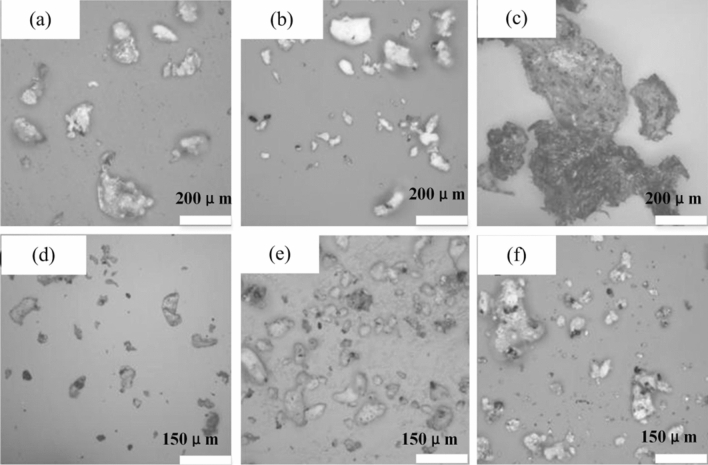
Particle morphology of PEKK and different types of graphene/PEKK composite materials with 1% graphene (200X): (**a**) PEKK; (**b**) GR_MG1_/PEKK; (**c**) GR_MG2_/PEKK; (**d**) GR_MG3_/PEKK (**e**); GR_MG4_/PEKK; (**f**) GR_XF001W_/PEKK (Visio 2021, Prof. WenPing. Song).

Figure 1 shows the microstructure morphology of the PEKK matrix and graphene/PEKK composite material (with 1% graphene content) powder under a stereo microscope magnified 200 times (SEM: S-3004N, Hitachi, Tokyo, Japan). In addition, the PEKK matrix, GR_MG1_/PEKK, GR_MG3_/PEKK, GR_MG4_/PEKK and XF_001w_/PEKK composite powders presented a granular distribution. GR_MG3_ and GR_MG4_ composite powders were relatively regular, and the particle size distribution was relatively concentrated, which is an important factor in hot pressing moulding. The particle size distribution of GR_MG2_/PEKK composite powder was dispersed, and significant aggregation occurred, which is an unfavourable factor for the moulding of graphene/PEKK composite powder. The agglomeration of the composite powder also indicated that the remaining oxygen-containing groups on GR_MG2_ were relatively high, which caused the agglomeration of graphene.

**Figure 2:**
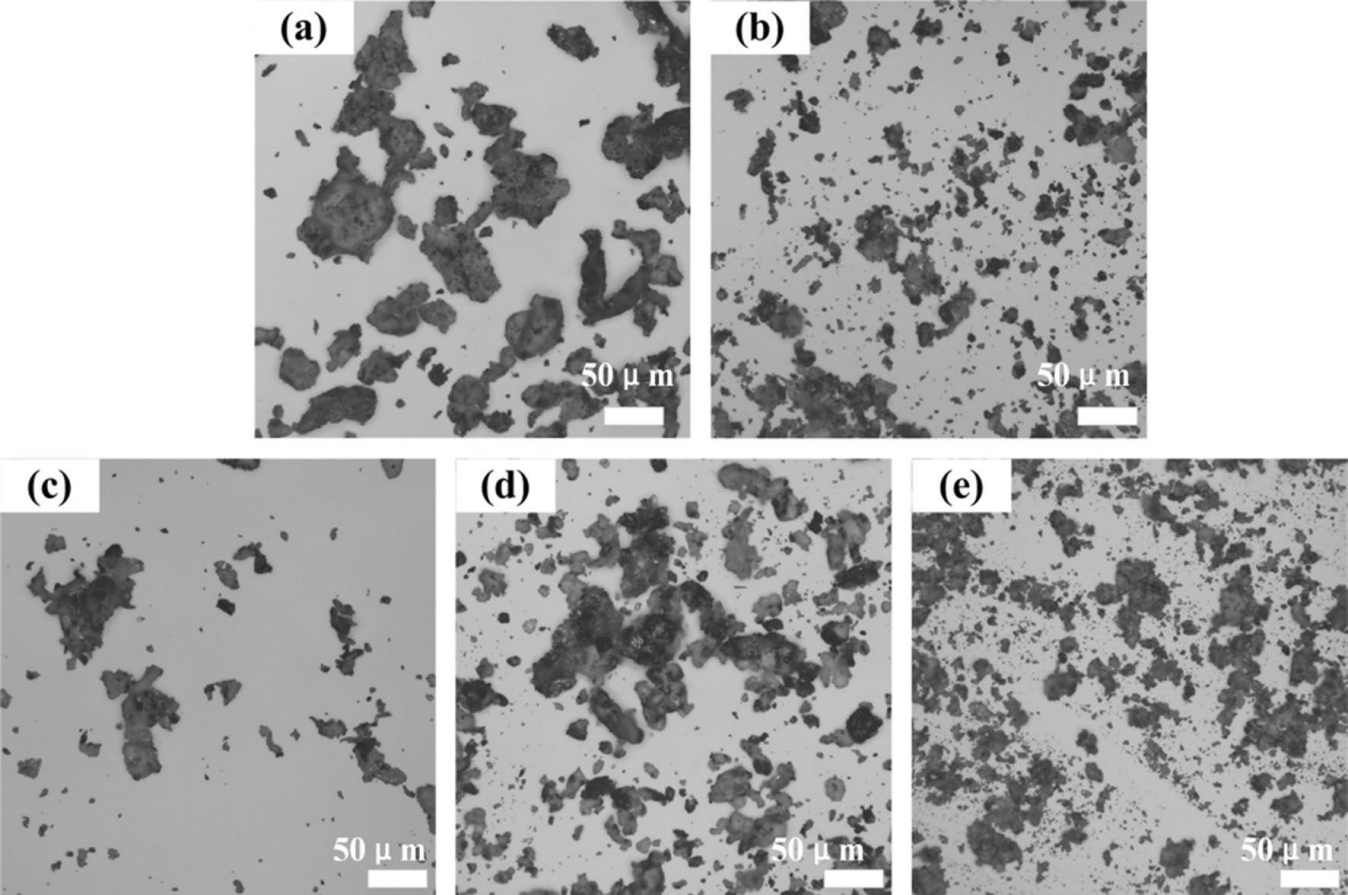
Dispersion of graphene in PEKK matrix: (**a**) 2%, (**b**) 3%, (**c**) 4%, (**d**) 5%, (**e**) 6% (Visio 2021, Prof. WenPing. Song).

Based on the above-mentioned discussion, GR_MG3_ powder will be selected as the experimental materials to prepare graphene_x_/PEKK composite materials. Figure 2 shows the dispersion of graphene in the PEKK matrix (the different contents of graphene) (SEM: S-3004N, Hitachi, Tokyo, Japan). The different contents of graphene/PEKK composite powder will be deformed to obtain graphene/PEKK composite sheets by hot pressing.

### Hot pressing process

**Figure 3:**
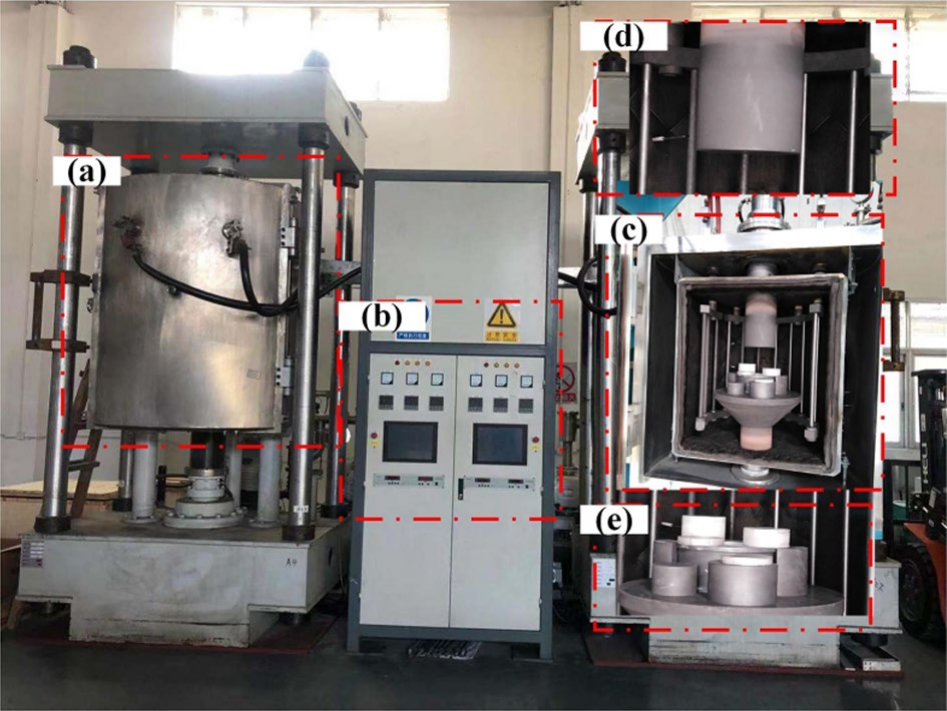
Vacuum hot pressing devices. (**a**) Vacuum hot pressing system; (**b**) Vacuum hot pressing control system; (**c**) Vacuum hot pressing furnace; (**d**) The punch; (**e**) Gaskets and indenters (Visio 2021, Prof. WenPing. Song).

High temperature is essential for composite base on PEKK, meanwhile, the oxidation of the graphene_x_/PEKK composite material is in contact with air during forming, and this condition affects the electrical conductivity of the graphene_x_/PEKK composite products. Thus, vacuum environment during hot pressing (Fig. 3) was particularly set to ensure conductive properties of the graphene_x_/PEKK composite. The equipment is divided into three parts, namely, vacuuming, heating and pressuring. The vacuum is always in working condition.

Figure 3a shows the vacuum hot pressing system. Samples were deformed under vacuum, high temperature and pressure. Figure 3b demonstrates the control system for controlling the temperature, pressure and stroke of the punch. Figure 3c shows the vacuum hot pressing furnace, along with the resistance, punch (Fig. 3d), gaskets and indenters (Fig. 3e). The equipment was used to obtain graphene/PEKK composite sheets with thickness of 1.2 mm and diameter of 80 mm. Although the sintering temperature was lower than the melting temperature at 270 °C and the carbonisation and oxidation of the graphene/PEKK composites were overcome during the moulding process, the thickness of the samples was limited. Moreover, in the central part of the unmelted graphene/PEKK composite powder, the powder was not uniformly heated, thereby requiring post-curing treatment. The equipment (Fig. 3) keep the pressure constant at 20 MPa. The flow state of the material is changed by temperature, and the thickness of the sheet is controlled by the stroke of the upper die to obtain the desired product.

Moreover, in the central part of the unmelted graphene/PEKK composite powder, the powder was not uniformly heated, thereby requiring re-curing treatment. Re-curing treatment was carried out in order to improve mechanical properties off graphene_x_/PEKK composite sheets further. The test specimens were re-cured at a heating rate of 4.83 °C/min and a cooling rate of 4.83 °C/min. The temperature was raised to 290 °C, held for 2 h and then dropped to room temperature at a rate of 4.83 °C/min to remove the part.

### Characterization

In order to determin sinter temperature of hot pressing process, the DSC test (DSC-NETZSCH STA 449F3, Selbu, Germany) was carried out between 0 and 450 °C at a heating/cooling rate of 10 ◦C/min for graphene/PEKK composite powders. Electronic universal testing machine (EUT504C, Shenzhen Tianyishi Technology Co. LTD) was used to investigate mechanical properties of the graphene/PEKK composite sheet, in addition, the conductivity of the composite sheet was characterized by Four-probe tester (SX1944, Suzhou Baishen Technology Co. LTD). The microtopography of the graphene/PEKK composite sheet was investigated with scanning electron microscopy (SEM) with S-3004N, Hitachi Ltd, Japan.

### Consent to publish

We agree to publish this article.

## Results and discussions

### Thermal properties of graphene/PEKK composite powder

**Figure 4:**
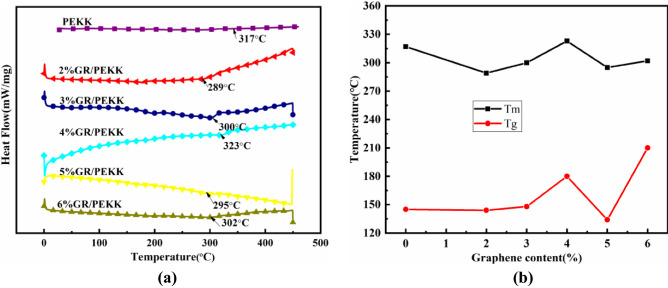
DSC curve and transition temperature and melting temperature of different graphene/PEKK composite materials: (**a**) DSC, (**b**) *T*_*g*_–*T*_*m*_*.*

The above-mentioned composite materials were processed and formed into electronic products and conductive part. The later sintering temperature of graphene/PEKK composite powders under hot pressing are designed according to their respective transition temperatures according to DSC test. The DSC curves, glass transition temperature and melting transition temperature of pure PEKK powder and graphene/PEKK composite powders with different contents are shown in Fig. 4 (Origin 2020).

The downward absorption peak in the DSC curve is called the melting peak. *T*_*g*_ refers to the glass transition temperature, which determines the softening temperature of the material. Graphene in the graphene/PEKK polymer composite caused *T*_*g*_ of the composite material to increase and decrease around *T*_*g*_ of the PEKK matrix. The graphene content increased to more than 3%, and *T*_*g*_ of the composite material changed around *T*_*g*_ of the matrix as the graphene content increased with the increase of *T*_*g*_. Graphene has high thermal stability, which improves *T*_*g*_ and *T*_*m*_ of the composite material. In addition, graphene was used as a conductive filler. With increasing graphene content, the damaging effect of graphene on the dense distribution of the matrix molecular segments increased (increased the movement ability of the chain segment). Therefore, graphene was the main factor in reducing *T*_*g*_ and *T*_*m*_ of composite materials.

*T*_*m*_ is a key parameter that determines the powder moulding temperature: graphene makes *T*_*m*_ of the composite material close to that of PEKK, but overall *T*_*m*_ of the matrix material is slightly lower. The addition of graphene increased the distance between molecular chains to a certain extent, improved the high mobility of the matrix segment, and destroyed the crystallization behaviour of the PEKK macromolecular segment, thereby lowering the melting point. Based on the above-mentioned experimental results, these different contents of graphene_x_/PEKK composite powders were used to obtain composite sheets using hot pressing pressure.

### Mechanical performance analysis of conductive graphene/PEKK composite sheet

Graphene/PEKK composite powders with different graphene contents were used as raw materials to obtain graphene-conductive samples by hot pressing. This paper focused on the mechanical properties of conductive graphene_x_/PEKK composite sheets molded by hot pressing. In addition, this study compared the microstructure morphology with the mechanical performance of graphene_x_/PEKK composite materials to determine the cause of the difference in mechanical behaviour. In this paper, the mechanical performance of PEKK and graphene (2%, 3%, 4%, 5%, 6%)/PEKK composites sheets obtained from hot pressing process was also discussed. Table 2 shows the performance parameters of different contents of graphene, including conductivity, melting temperature (*T*_*m*_), glass transition temperature (*T*_*g*_) and sintering temperature. The conductivity of the composite material is necessary.

**Table 2:** The performance parameters of different graphene content.

No	Context	Maximum conductivity(S/n)	T_m_ (°C)	T_g_ (°C)	Sinter temperature (°C)
1	PEKK	0	317	145	177
2	2%Graphene	0	289	144	177
3	3%Graphene	0.001	300	148	210
4	4%Graphene	0.009	323	205	210
5	5%Graphene	0.164	295	134	210
6	6%Graphene	0.126	302	210	240

Sintering temperature is an important factor affecting hot pressing moulding quality. A low sintering temperature cannot easily melt the composite powder, whereas a high temperature can easily cause decomposition of the PEKK matrix. On the basis of the *T*_*m*_ and *T*_*g*_ of graphene/PEKK composite powders, the range of sintering temperature of hot pressing for these composite powders can be determined. During hot pressing, the powder in the mould is heated to the sintering temperature, and the composite powder presents an approximate fluid state, and finally cools and solidifies into the desired shape.

**Figure 5:**
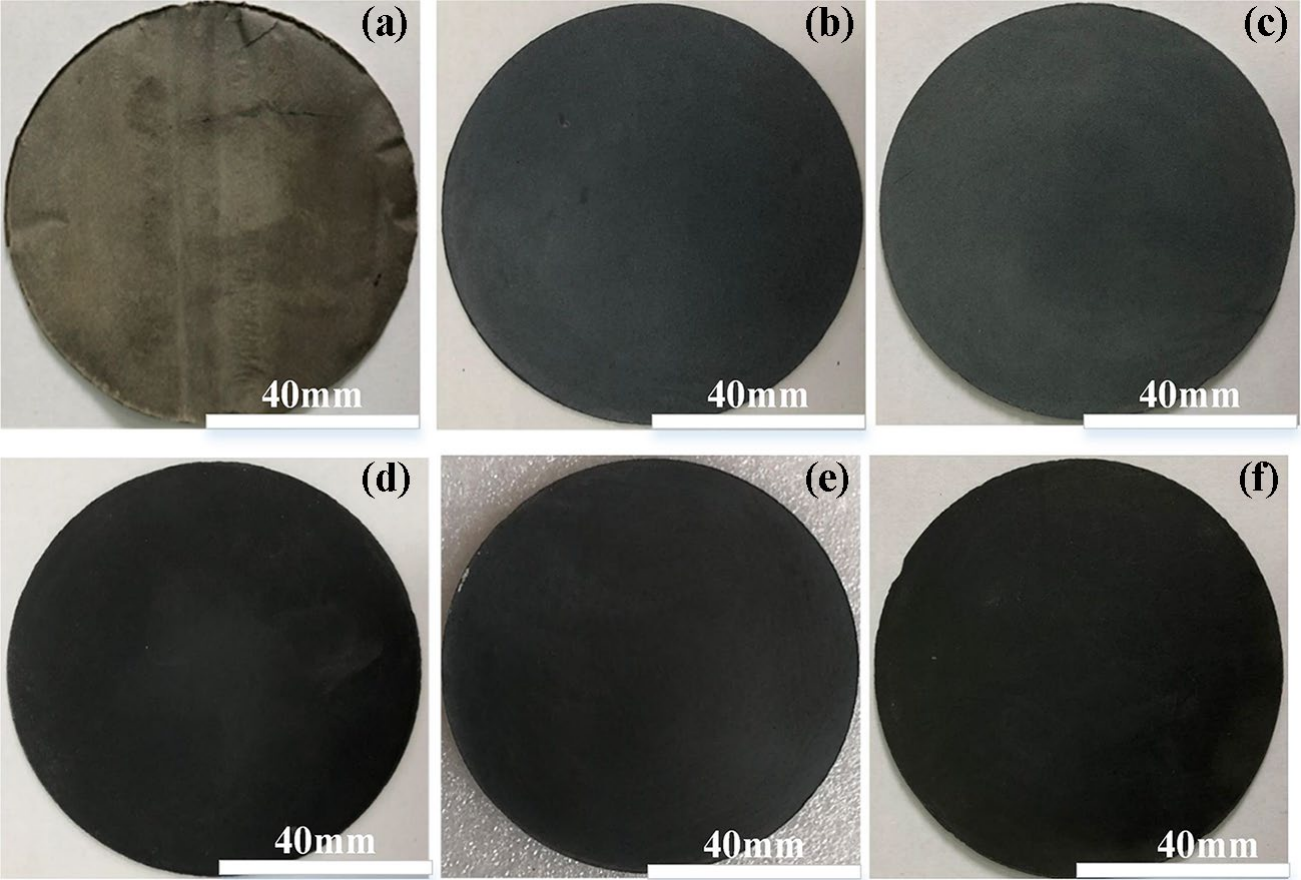
Sheets with diameter 80 mm and thickness 1.25 mm. (**a**) Graphene = 0%; (**b**) graphene = 2%; (**c**) graphene = 3%; (d) graphene = 4%; (**e**) graphene = 5%; (**f**) graphene = 6% (Visio 2021, Prof. WenPing. Song).

PEKK and graphene_x_/PEKK (x = 2%, 3%, 4%, 5%, 6%) were moulded by vacuum hot pressing (Fig. 3) to obtain circular sheets of 80 mm diameter and 1.25 mm thickness (Fig. 5). The sintering temperature was set between the glass transition temperature and melting temperature. It was normally set at the middle of the melting temperature and glass transition temperature, and the deviation did not exceed 30 °C. Pressure is also an important factor influencing the mechanical properties and density of graphene/PEKK composite sheet. In this research, pressure is 20 MPa (Fig. 3).

**Figure 6:**
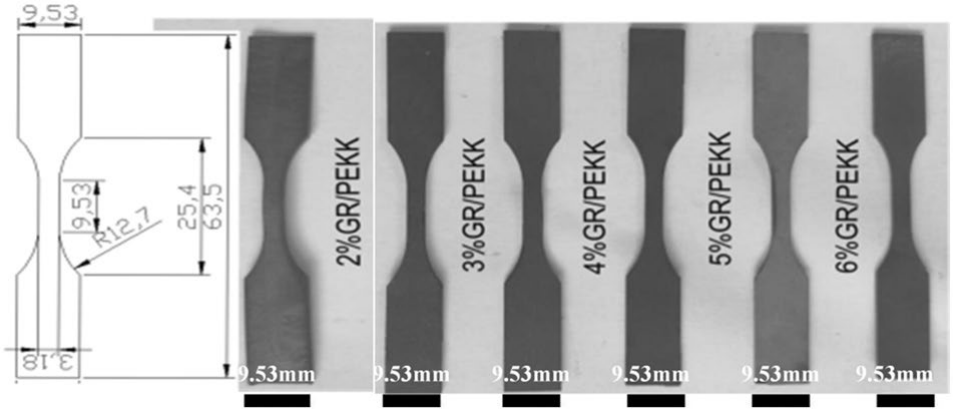
Tensile specimens for the different graphene content (Visio 2021, Prof. WenPing. Song).

The graphene/PEKK composite sheets were cut by laser on the basis of the standard samples (Fig. 6). The specimens shown in Fig. 6 were obtained from PEKK and graphene_x_/PEKK composites sheets, and the specimen was sandpapered after cutting to avoid failure caused by burrs. The tensile tests were carried out with a stretching rate of 5 mm/min and a tensile gauge spacing of 10 mm.

**Figure 7:**
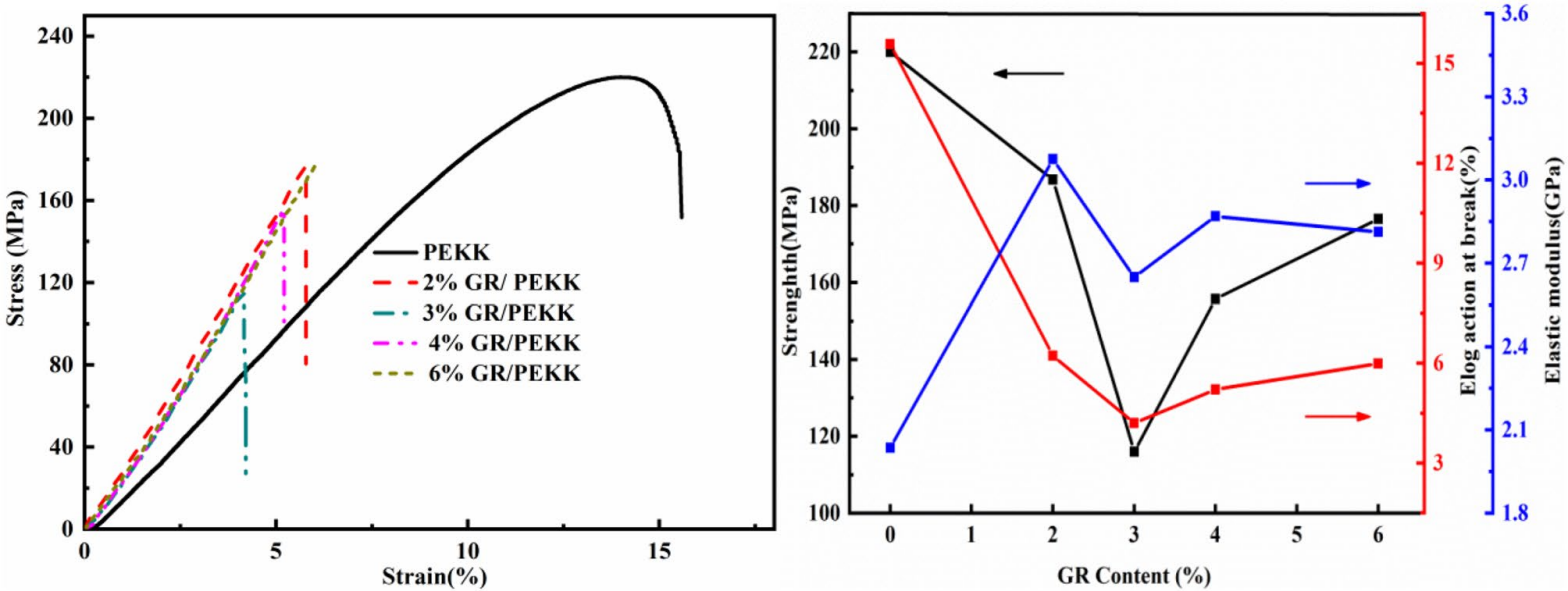
Tensile measurement for PEEK and graphene_x_/PEEK composites: (**a**) stress–strain curves from uniaxial tensile tests, (**b**) performance of composite materials.

Figure 7a shows the stress–strain curve of the graphene/PEKK composite stretched at room temperature (Origin 2020). As shown in Fig. 7a, the fracture mode of the PEKK matrix is a strong toughness fracture, whereas that of the graphene_x_/PEKK composite material is a typical brittle fracture. Figure 7b shows the tensile strength, elastic modulus and elongation at break of the graphene_x_/PEKK composite (Origin 2020). As shown in Fig. 7b, Young’s modulus of the pure PEKK sample is 2.04 GPa; the tensile strength is 220.17 MPa, and it finally breaks at a strain of 15.6%. Compared with pure PEKK, the strength and elongation at break of the composite materials generally decrease, and the elastic modulus generally increases. With the increase of graphene content, the strength, elastic modulus and elongation at break of the composite material decrease initially and then increase, and they are the lowest at graphene content of 3%, respectively. For the graphene_x_/PEKK composite material, the tensile strength ranges from 116 to 186 MPa, the elastic modulus ranges from 2.6 to 3.6 GPa, and the value range of elongation at break is from 4 to 6%. It should be noted the mechanical properties of graphene_x_/PEKK composite with highest graphene content (6%), which shows a strength of 177 MPa, an elastic modulus of 2.8 GPa, and an elongation at break of 6%.

PEKK is a semi-crystalline polymer, and its high strength depends on the crystalline structure inside the material to a large extent. Graphene destroys the regular arrangement of PEKK molecular chain segments to a certain extent and reduces its crystallinity. Thus, the strength of the prepared composite material is reduced. In addition, given the lamellar microstructure and powdery macroscopic state of highly rigid graphene in the prepared composite material, the network structure of the material is destroyed to a certain extent after it enters the PEKK material matrix, which directly leads to the material. The brittleness is increased, and stress concentration easily occurs, thereby damaging the material and decreasing tensile strength. Graphene improves the elastic modulus of the composite material probably because of its high modulus. It enters the network voids of the PEKK polymer matrix and serves as a skeleton, which can still be used to a certain extent. The elastic modulus of the material is also improved.

The strength results show that graphene_x_/PEKK composites prepared by situ polymerization and molded by hot pressing in this paper have a higher elastic modulus and tensile strength of approximately 40 MPa compared with the 10% nano-graphene composites discussed in previous literature^24^. However, previous studies and the present study clearly show a decrease in toughness of the composites with the addition of graphene, thereby indicating that with the increase of carbon, the toughness of the material decreases, but the stiffness increases.

### Morphology of graphene/PEKK composite sheets

**Figure 8:**
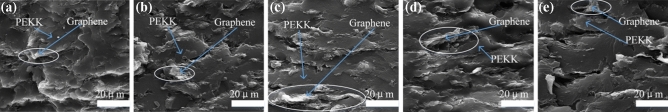
SEM analysis for the different graphene content composite sheets: (**a**) 3%; (**b**) 4%; (**c**) 4.5%; (**d**) 5%; (**e**) 6% (Visio 2021, Prof. WenPing. Song).

Figures 8 shows the microscopic morphology of graphene/PEKK composite sheets with different graphene contents (graphene context is more than 3%, with conductivity) after brittle fracture, which is obtained by SEM (SEM: S-3004N, Hitachi, Tokyo, Japan ). Above the percolation threshold, no graphene-aggregated structure is observed in the cross-sectional morphology of the composite material with different graphene contents. Therefore, graphene has a good dispersion uniformity in the composite material.

Compared with (b), (c), (d) and (e), the graphene sheet shown in Fig. 8a has a low number of superimposed layers, indicating that the strength is weaker than the other four types, and the conductivity is not as good as the other composite with different graphene contents. For the low content of graphene and pure PEKK composite materials, 3% graphene neither forms a conductive path nor serves as a reinforcing substrate. As the content of graphene increases, the layered structure of the cross-sectional morphology of the composite material becomes increasingly evident (Fig. 8d,e), which is primarily due to the stacked structure of graphene with folded sheets to compound with the PEKK matrix. Therefore, the experimental results of SEM show that the in situ polymerization achieves not only the uniform distribution of graphene sheets in the matrix, which overlap with one other in different orientations, by forming a conductive path to achieve the conductive properties of the composite material, but also the strong interface bond between the filler graphene and PEKK substrates and the good compatibility between the two kinds of materials.

## Conclusion

Based on situ polymerization of graphene_x_/PEKK composite powder with different graphene contents, pressing moulding of graphene_x_/PEKK composite powders was explored by using vacuum hot pressing equipment to realize conductive property and good mechanical properties of the graphene/PEKK composites sheets with thickness of 1.25 mm and diameter of 80 mm.

The vacuum environment and molding temperature were particularly designed in order to obtain high conductive and good mechanical properties for the graphene/PEKK composite sheet. The vacuum environment ensure that the composite powder is not oxidized and then lose electrical conductive property; the designed molding temperature was between *Tg* and *Tm* of each graphene/PEKK composite according to DSC tests, which are crucial for the quality of the molded product, especially for mechanical properties.

In addition, compared with the elastic modulus of the PEKK polymer sheet, the elastic modulus of the graphene_x_/PEKK composite was higher than 50%, although its toughness was less than 10%. The composite sheet with a graphene content equal to or above 3wt% showed good conductive property. The SEM microscopic morphology of graphene_x_/PEKK composite sheets with different graphene contents showed that graphene were satisfactory dispersed in the PEKK matrix. This experimental study provided a practical solutions for molding of graphene/PEKK composite powder for achieving high conductive properties and good mechanical properties.

## Abbreviations

PAEK: Polyaryletherketone
PEKK: Poly(ether-ketone-ketone)
PEEK: Poly(ether-ether-ketone)
DSC: Differential scanning calorimetry
SEM: Scanning electron microscopy
